# The emerging role of β-secretases in cancer

**DOI:** 10.1186/s13046-021-01953-3

**Published:** 2021-04-29

**Authors:** Francesco Farris, Vittoria Matafora, Angela Bachi

**Affiliations:** IFOM- FIRC Institute of Molecular Oncology, Milan, Italy

**Keywords:** BACE1, BACE2, Amyloid aggregates, Tumor microenvironment, Cancer

## Abstract

BACE1 and BACE2 belong to a class of proteases called β-secretases involved in ectodomain shedding of different transmembrane substrates. These enzymes have been extensively studied in Alzheimer's disease as they are responsible for the processing of APP in neurotoxic Aβ peptides. These proteases, especially BACE2, are overexpressed in tumors and correlate with poor prognosis. Recently, different research groups tried to address the role of BACE1 and 2 in cancer development and progression. In this review, we summarize the latest findings on β-secretases in cancer, highlighting the mechanisms that build the rationale to propose inhibitors of these proteins as a new line of treatment for different tumor types.

## Background

### BACE1 and BACE2: an overview

**Fig. 1:**
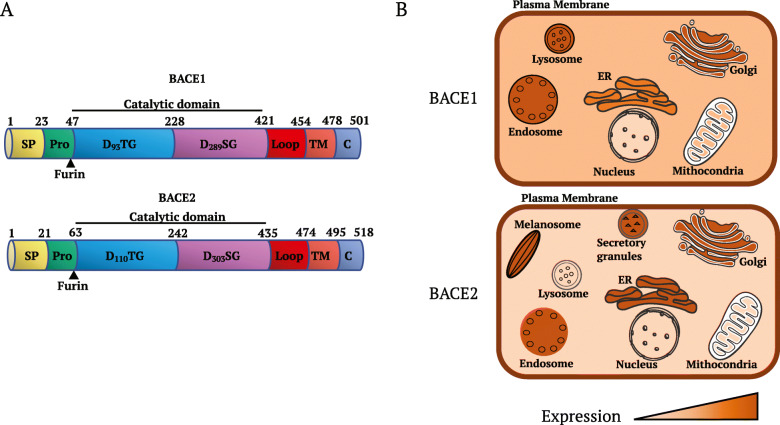
Structural organization and subcellular localization of BACE1 and BACE2. SP = signal peptide; Pro = pro-domain; DTG, DSG = catalytic domains; TM = transmembrane domain; C = C-terminal domain.

BACE1 and BACE2 (β-site APP cleaving enzyme 1 and 2) are membrane glycoproteins that act as aspartic proteases. Both BACE1 and BACE2 are synthetized in the ER (endoplasmic reticulum) [1]; structurally, they are formed by a signal peptide (SP), a pro- domain, a core protease domain, a transmembrane domain and the C-terminal domain [2] (Fig. 1 a). After being synthetized, these enzymes move to the Golgi where the Furin convertase cleaves the pre- and the pro- domains to produce a fully active enzyme capable to process its substrates [2–5]. In particular, the core protease domain has a classical aspartic protease motif (DTG, DSG) that allows the shedding of substrates’ ectodomains [6] releasing them in the extracellular space [7].

BACE1 is highly expressed in the central nervous system, while BACE2 does not have an organ specific expression [8]. Both proteins are membrane-anchored and have been localized in the later Golgi/transGolgi network, on the cell surface membrane and on the membrane of intracellular vesicles (Fig. 1b) [9]. In addition, BACE2 localize on the secretory granules of pancreatic β-cells [10] and on early-stage melanosomes in melanocytes [11].

The shedding activity of BACE1 and BACE2 has different physiological and pathological functions that we will summarize in the next chapters.

### BACE1 physiological targets

BACE1 has been studied mostly in the central nervous system, where it takes part in the processing of the amyloid-beta precursor protein (APP). Specifically, BACE1 cleaves APP at the β-site between the aminoacids Met-671 and Asp-672 [6, 12–15], generating sAPPβ (soluble amyloid precursor protein β), which as a role in synapses pruning during the nervous system development and glia differentiation [16, 17], and a C99 fragment that, after being cleaved by γ-secretase, produces the Amyloid Beta (Aβ) peptides [18]. BACE1 is also able to cleave other proteins belonging to the APP family such as APLP2 (Amyloid-like protein 2) [19, 20]. In this case, BACE1 processes APLP2 generating an N-terminal fragment and a short C-terminal fragment [20]. Not much is known about APLP2 physiological role but it seems to be involved in the differentiation of neural precursor cells during nervous system development [19, 21]. Beyond its role in processing APP and APP family members, BACE1 acts on other substrates involved in nervous system homeostasis [22]. Indeed, BACE1 is implicated in myelination during development and remyelination in adulthood through the processing of Nrg1 (Neuregulin 1) [23]. Specifically, the loss of BACE1 reduces Nrg1 shedding and the activation of the Akt signaling pathway that controls the expression of genes linked to myelinization [24]. Moreover, the missed cleavage of Nrg1 reduces the expression of transcription factors belonging to the early growth response family, responsible for the expression of muscle-spindle specific genes involved in the formation of muscle spindle fibers [25]. It has also been reported that, in the context of central nervous system development, BACE1 cleaves Jagged-1 (Jag1), a Notch ligand, counteracting the activation of the Notch pathway in order to balance neurogenesis and gliogenesis [26]. Actually, it has been demonstrated that silencing of BACE1 brings to Notch pathway hyperactivation in neural stem cells, promoting differentiation towards astrocytes and reducing neurogenesis [26].

### BACE2 physiological targets

**Fig. 2:**
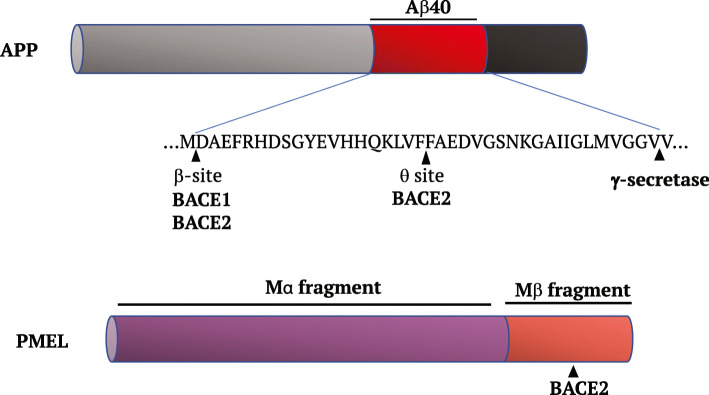
BACEs processing of APP and PMEL.

As its homolog, also BACE2 is able to cleave APP but in a different way. Indeed, different studies claim that BACE2 behave more like an α-secretase on APP [19, 27]. In particular, Farzan at al. showed that BACE2 cleaves APP not only in the same site of BACE1 but also at θ site, between Phe19 and Phe20 of the Aβ peptide forming a shorter, not neurotoxic Aβ peptide [27] (Fig. 2).

Outside the central nervous system, BACE2 has a well characterized role in maintenance of pancreatic β-cells [22]. In particular, BACE2 has been identified as the sheddase of TMEM27 (Trimeric intracellular cation channel 27). TMEM27 is important to maintain β-cells mass and to control glucose homeostasis. It has, in fact, been demonstrated that mice lacking BACE2 show high β-cell mass and improved glucose tolerance, suggesting that BACE2 could be a new target to maintain glucose homeostasis in diabetic patients [28]. Another BACE2 target, implicated in type 2 diabetes (T2D), is hIAPP (human Islet Amyloid Polypeptide) [29–31] an amyloidogenic protein, hallmark of T2D. BACE2 cleaves the mature hIAPP at Phe15 and Phe23, thus decreasing the cytotoxic fibrils formed by hIAPP [32]. This evidence seems to be in contrast with findings that advocate BACE2 inhibition as a way to stabilize TMEM27 with beneficial effect on β-cells mass and improved glucose homeostasis [28].

Finally, a study by Alcarraz-Vizan et al. claims that blocking BACE2 enzymatic activity in cells transfected with hIAPP improves insulin secretion after glucose stimulation, suggesting that the inhibition of BACE2 has the capacity to improve glucose homeostasis despite the formation of hIAPP amyloid fibrils [33].

Another well characterized physiological target of BACE2 is PMEL (premelanosome protein), a melanocyte specific protein, involved in the formation of an amyloid fibrils’ matrix fundamental for the deposition of eumelanin in melanosomes [11, 34, 35]. BACE2 cuts PMEL within the Mβ fragment to release the luminal amyloidogenic Mα fragment (Fig. 2) [36, 37]. Indeed, in BACE2 Knock out mice, Mβ and Mα PMEL fragments do not break apart and this missed cleavage does not allow the formation of PMEL amyloid fibrils determining a defect in pigmentation [11].

In addition, BACE2 plays a role in the development of melanophores. It has been reported that zebrafish larvae with homozygous mutation of BACE2 show dilated melanophores which have an abnormal migration pattern [38].

Further, Sez6L and Sez6L2 were identified as BACE2 targets in pancreatic cells but as BACE1 targets in brain, opening the possibility of a substrate’s selectivity based on the relative expression levels of the two enzymes [39].

### Pathological involvement of β-secretases

Despite BACE1 and BACE2 were discovered simultaneously [2], BACE1 captured first the attention of the scientific community mainly for its role in regulating the formation of cytotoxic Aβ peptides in Alzheimer's Disease (AD) [40]. For the same reason, BACE1 inhibitors have been developed to counteract the biogenesis of toxic amyloid beta peptides. Some of these drugs have been investigated in clinical trials but, despite reducing the production of Aβ peptides, unfortunately, they did not rescue the cognitive defect of AD patients [40, 41].

On the other hand, BACE2 has been targeted only more recently, in particular because it has been found to be involved in type 2 diabetes and upregulated in a broad range of tumors where its activity has been correlated with tumor progression.

This review has the goal to revise the recent literature about the link between β-secretases expression and molecular mechanisms involved in cancer growth and progression.

### Expression of BACE1 and BACE2 in cancer

In 2000, Xin et al. noticed, through gene expression profiling, that BACE2 was differentially expressed in human breast cancer cell lines, being upregulated in highly tumorigenic and metastatic cells [42]. Four years later, another study by Tsuji and colleagues, reported the upregulation of BACE2 in colon adenocarcinoma compared to normal tissue [43]. Both studies describe an upregulation of BACE2 in an aggressive and advanced stage building a correlative link between BACE2 and cancer progression.

**Fig. 3:**
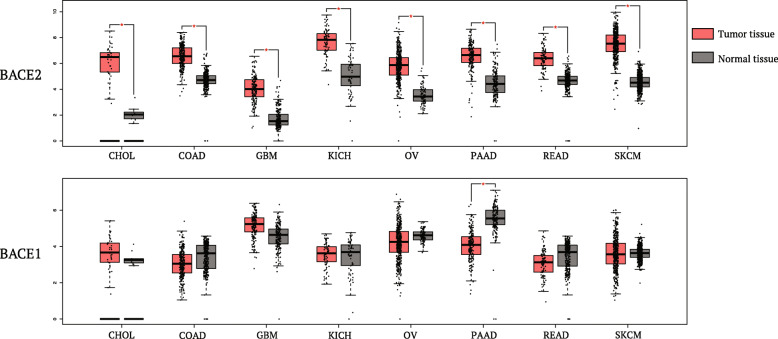
BACE1 and BACE2 expression profiling by cancer type. The gene expression profile across all tumor samples (red boxes) and paired normal tissues (grey boxes) is reported. Each dot represents the level of expression/ sample. CHOL (Cholangiocarcinoma); COAD (Colon adenocarcinoma); GBM (Glioblastoma); KICH (Kidney Chromophobe); OV (Ovarian serous cystadenocarcinoma); PAAD (Pancreatic adenocarcinoma); READ (Rectum adenocarcinoma); SKCM (Skin Cutaneous Melanoma).

Very recently, we and other groups showed an upregulation of BACE2 also in melanoma [44] and in glioma [45, 46]. Indeed, by interrogating GTEx (Genotype-Tissue Expression) and TCGA (The Cancer Genome Atlas) mRNA expression datasets through GEPIA (Gene Expression Profiling Interactive Analysis) [47], it is evident that BACE2 is upregulated in a broad range of tumors (Fig. 3) while BACE1 does not appear to be dysregulated in cancer.

**Fig. 4:**
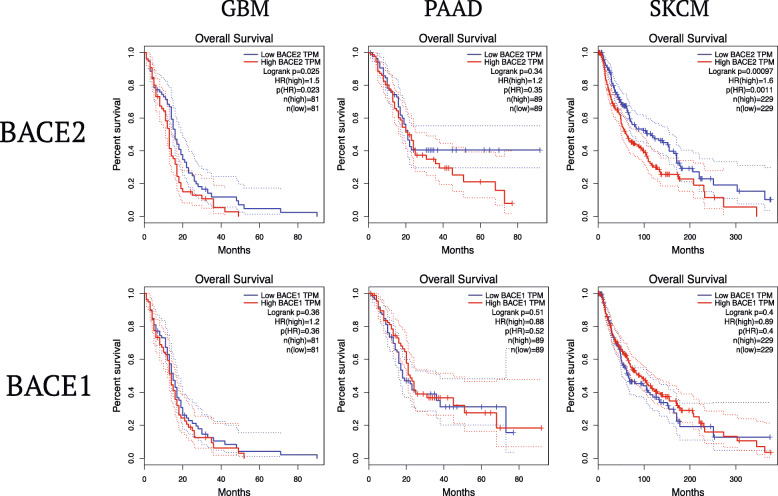
Kaplan-Mayer survival curve analysis. The solid line represents the survival curve, and the dotted line represents the 95 % confidence interval. Log-rank *P* < 0.05 is considered to indicate a statistically significant difference. Patients with expression above the median are indicated by red lines, and patients with expression below the median are indicated by blue lines. GBM (Glioblastoma); PAAD (Pancreatic adeno-carcinoma); SKCM (Melanoma).

Importantly, high BACE2 expression correlates with worse prognosis in melanoma [44, 48] pancreatic cancer [48] and glioma (Fig. 4), implicating an active role of this protease in cancer progression. As expected, BACE1 expression does not correlate with the prognosis of the disease (Fig. 4) pointing out that BACE2, and not BACE1, seems to be mainly involved, directly or through its targets, in the pathogenesis and/or progression of cancer.

### Implication of BACE1/2 targets in cancer

One of the first study that implicated BACE1/2 in tumor development was conducted in pancreatic cancer where Peters et al. noticed an upregulation of APP and APLP2 in tumor tissues compared to the normal ones. For this reason, they tried to inhibit BACE1/2, with different BACE1/2 inhibitors, noticing a reduction of cell growth and viability [49]. This important observation led to the hypothesis that APP and APLP2 soluble fragments may act on cellular pathways that modulate cell growth.

Notably, APP appears to play a crucial role on proliferation of tumor cells also in breast and colon cancer [50–53]. In particular, two different publications addressed the role of sAPP as driver of the proliferative phenotype in these two types of tumors, demonstrating that silencing of APP results in a reduced proliferation capacity of tumor cells. Moreover, the administration of conditioned media from parental cells or of sAPP can rescue proliferation of APP-silenced breast or colon cancer cells. These experiments highlight the crucial role of APP processing by beta secretases in cancer [53, 54] but the molecular mechanism underlining this effect still has to be elucidated.

### BACE1/2 driven mechanisms and cancer progression

In the last decade, a very active field in cancer research has been the study of tumor microenvironment (TME) [55–57]. The tumor microenvironment is composed of immune cells, such as macrophages, lymphocytes and neutrophils, of endothelial and stromal cells, of the extracellular matrix (ECM) and of soluble factors [58]. Inside the microenvironment, the soluble factors, secreted by tumor cells, are able to recruit all these different types of cells, that, educated by the tumor itself, produce a pro-tumoral environment.

For example, macrophages, one of the most represented categories of cells in the tumor microenvironment, are recruited by different soluble factors produced by the tumor, such as CSF1 (colony stimulating factor 1**)** and IL6 (Interleukin-6). When recruited in TME, macrophages produce IL10 (Interleukin-10), TGFβ (transforming growth factor-β) and VEGF (vascular endothelial growth factor) creating an immunosuppressive environment and participating in neoangiogenesis and metastatization [59, 60]. Other dynamic players in TME are cancer associated fibroblasts (CAFs) that, activated by the tumor, secrete different ECM components [61], growth factors and cytokines creating an environment permissive for tumor growth and promoting an ECM stiffening that constitute a “safe heaven” for the tumor cells where they can proliferate and acquire drug resistance [62]. This interplay between non-tumor cells and cancer cells highlights a functional crosstalk capable of affecting both the biology and the progression of the disease.

As tumor microenvironment is shaped by different soluble factors, β-secretases, thanks to their ability of shedding polypeptides in the extracellular space, can be considered key players that orchestrate the crosstalk between the tumor and the surrounding cells.

### Effect of BACE1/2 on TME

One of the first evidences suggesting BACE1/2 as pro-tumorigenic enzymes come from the observation that the inhibition of these proteins reduces both the proliferation of endothelial cells and the formation of capillary structures in vitro. These findings were also confirmed *in vivo*, where the administration of BACE1/2 inhibitors results in a decreased tumor volume of xenotransplanted glioblastoma and lung adenocarcinoma and impacts negatively on tumor vascularization [63].

**Fig. 5:**
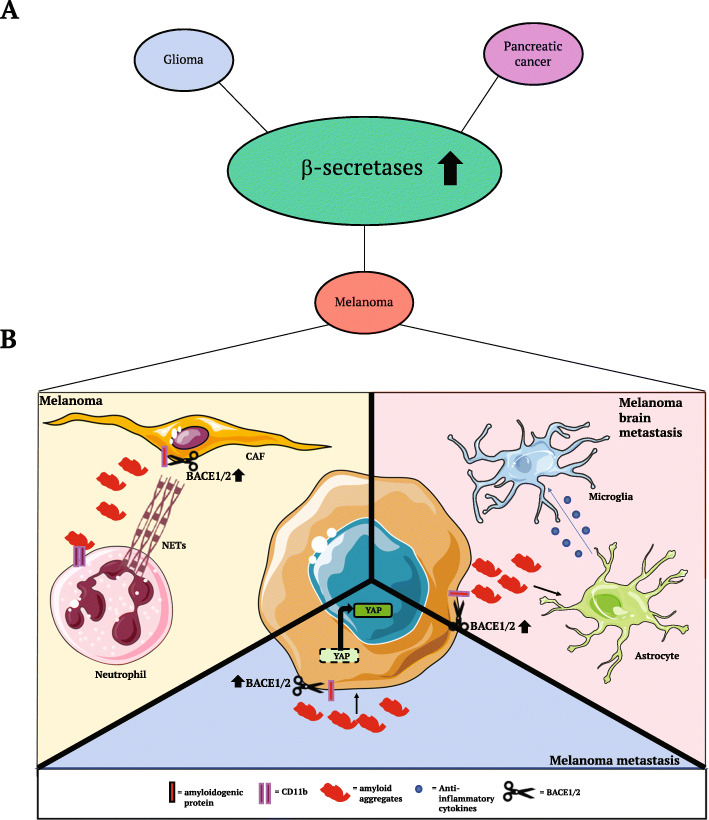
BACE1/2 in cancer. BACE1/2 are overexpressed in pancreatic cancer, glioma and melanoma (**a**) and specifically they are involved in the processing of amyloidogenic proteins modulating TME of melanoma (**b**)

Very recently, it has been reported that BACE1/2 derived maturation of amyloid beta can drive NETs (Neutrophil Extracellular Traps) deposition in pancreatic cancer and in melanoma [48]. NETosis, the release of NETs by neutrophils, is a physiological defense mechanism, occurring when neutrophils extrude in the extracellular space decondensed chromatin which has a microbicidal effect [64]. It is known that Amyloid beta can induce NETosis in AD [65, 66] where it exacerbates neuroinflammation, promoting vascular and parenchyma damage. In addition, cancer cells can promote NETosis, which in turn promotes metastasis formation by trapping tumor cells and facilitates cancer progression [67, 68]. Munir and colleagues made the interesting observation that NETosis, in melanoma and pancreatic cancer biopsies, is clearly evident in areas populated by CAFs. They also discovered that the secretome of CAFs, in comparison with the secretome of normal fibroblasts, is particularly enriched in APP and amyloid beta peptide [48]. Moreover, they observed a strong reduction of amyloid beta by treating CAFs with a dual β-secretase inhibitor with the consequence that supplementing neutrophils with this amyloid beta-depleted secretome strongly reduces NETosis. To demonstrate that this effect is driven by amyloid beta, and not by other BACE targets, they administered the recombinant amyloid beta peptide together with CAFs conditioned medium lacking endogenous amyloid beta and noticed a dose-dependent rescue of NETosis (Fig. 5b) [48]. In vivo, the administration of conditioned media from CAFs, or of recombinant amyloid beta, increased systemic NETosis in mice. Having demonstrated that NETosis can be modulated by the β-secretases activity on APP, they wondered how this process influences tumor growth and observed that inhibiting BACE1/2 in skin tumor bearing mice results in a strong reduction of tumor volume [48]. These experiments clearly point to BACE1/2 as a potential therapeutic target in melanoma and pancreatic adenocarcinoma.

A second study, underlining the relevant role of BACE1/2 activity in cancer, describes a paracrine effect of amyloid beta in promoting melanoma brain metastases [69]. In this case, it has been shown that melanoma derived brain metastases produce the Aβ40 peptide which, through the cross-talk with astrocytes, induces an immunosuppressive environment that foster brain colonization by melanoma cells (Fig. 5b) [69]. In details, the authors silenced APP in melanoma cells without affecting melanoma proliferation *in vitro* but noticed a reduction in brain metastases formation *in vivo* in a melanoma mouse model. In the same study, they also use a dual BACE1/2 inhibitor to block the production of Aβ in vivo, demonstrating the efficacy of this kind of treatment in reducing the number of brain metastases and tumor burden and suggesting BACE1/2 inhibition as new therapy against melanoma progression [69].

Recently, the BACE2 processing of amyloidogenic proteins, has also been suggested to affect tumor proliferation in a cell autonomous fashion [44]. In particular, a study from our laboratory identified proteins differentially secreted between primary and metastatic melanoma cells observing an enrichment of amyloidogenic proteins known to be BACE1/2 targets, such as APP, APLP2 and PMEL, in the metastatic secretome. Interestingly, amyloid fibrils were found specifically enriched not only in metastatic melanoma cell lines but also in vivo in human melanoma biopsies.

Mechanistically, we showed that amyloid fibrils, and in particular the PMEL Mα fragment, impact on YAP (Yes Associated Protein) transcriptional activity thus sustaining melanoma proliferation. Moreover, we hypothesized that, amyloids, through their exceptional rigidity [70], might be able to modulate the ECM stiffness activating mechanotransduction (Fig. 5b). Further, we demonstrated that BACE2 activity is required not only to produce amyloid fibrils but it also affects melanoma cells sensitivity to chemotherapy [44].

Coherently, it has also been observed that APP knock down in melanoma cells [71] and in pancreatic cancer [72] enhanced the cytotoxicity of different chemotherapeutic agents, indicating that the presence of amyloid fibrils can indeed modulate response to drugs.

Another study that describes the activity of BACE in TME, found that Verubecestat, a BACE1/2 inhibitor [73] promotes an increase in glioma phagocytosis mediated by macrophages, thus hampering tumor growth. In details, Zhai et al. found that BACE1 is overexpressed in glioma infiltrating tumor associated macrophages (TAM) compared to normal macrophages. They also demonstrated that BACE1 induces a pro-tumoral phenotype of TAM through the shedding of sIL6R (soluble IL-6 receptor) that forms a complex with IL-6, in the extracellular space, driving STAT3 activation, a crucial pathway for macrophages pro-tumoral activation. On the other hand, BACE1 inhibition promotes a switch from pro-tumoral macrophages (pTAM) to tumor suppressive macrophages (sTAM). In addition, they showed that low dose radiation is able to increase TAMs infiltration and that a concomitant Verubecestat administration converts pTAM in sTAM, reducing tumor growth and demonstrating that low dose radiation therapy synergizes with BACE inhibition [73].

### The role of BACE2 in cancer through intracellular pathways

Beside the role in processing and shedding proteins that modify the TME, β-secretases activity has also been shown to have a pro-tumorigenic function by modulating intracellular pathways.

For instance, the pro-tumoral effect of BACE2 has been observed in glioma [45, 46] and ocular melanoma [74]. In glioma, BACE2 has been shown to increase migration and invasion by inducing an epithelial to mesenchymal transition. Actually, it has been observed that BACE2 silencing results in a downregulation of epithelial markers and in an upregulation of mesenchymal markers. Moreover, BACE2 hyperactivates NF-κB (nuclear factor kappa-light-chain-enhancer of activated B cells) pathway, through a series of phosphorylation cascade of different member of this pathway, such as p65, IKKβ (inhibitor of nuclear factor kappa-B kinase subunit beta) and IKBα (nuclear factor of kappa light polypeptide gene enhancer in B-cells inhibitor, alpha), increasing tumor growth [46]. Further, BACE2 silencing in glioma was shown to decrease tumor volume in mice and to increase the effect of radiation therapy [45].

Another remark of the connection between BACE2 activity and modulation of intracellular pathways to support cancer growth comes from a study on ocular melanoma. In this disease, BACE2 has been linked to an increased expression of TMEM38B (Trimeric intracellular cation channel 38b), a calcium channel, leading to Ca^2+^ intracellular accumulation and activation of the PI3K (Phosphoinositide 3-kinase) pathway that sustain tumor proliferation [74].

Taking together all these observations, it is becoming more and more evident that BACE2 plays a crucial role in promoting cancer growth and progression, either via modulation of the microenvironment or by a cell autonomous mechanism, reinforcing the idea of exploiting BACE1/2 as a new target for cancer therapy.

## Discussion

BACE1 and BACE2 are two aspartic proteases involved in ectodomain shedding of different substrates. Despite their high grade of homology, they have distinct functions depending on the cellular context. BACE1 plays a role mostly in the central nervous system while BACE2 is mainly involved in β-cells maintenance and melanocytes pigmentation [2]. These distinct roles reflect their tissue distribution, as BACE1 is highly expressed in different regions of the nervous system and BACE2 is broadly distributed in peripheral tissues [8].

These two proteases, for their function in processing APP into amyloid beta fibrils, have been deeply studied in Alzheimer's disease. In particular, BACE1 plays a major role in the formation of Aβ neurotoxic peptides which then form amyloids plaques [6, 12]. The role of BACE2 is instead controversial: on one side, BACE2 can cleave APP at theta site forming a shorter, less toxic, Aβ peptide compared to the one derived from BACE1 processing [27]; on the other side, BACE2 expression and function have been correlated with an increased neurodegeneration in Alzheimer 's disease [75, 76]. Several studies assess an involvement of BACE2 in type-2 diabetes, where BACE2 inhibition increase β-cell mass favoring glucose homeostasis [28, 33].

Only in the last 2 years, BACE1/2 have been implicated in cancer progression. Indeed, BACE2 is highly expressed in a broad range of tumor tissues and its expression is associated with worse prognosis (Figs. 3 and 4).

The first indication that β-secretases might be involved in cancer came from the observation that BACE2 are highly expressed in breast cancer and colon adenocarcinoma. These studies built a correlation between the level of expression and the stage of the disease without indicating a direct link or a potential mechanism [42, 43].

More recently, BACE2 have been reported to be highly expressed also in other types of tumors, such as glioma [45, 46] and melanoma [44], where different mechanisms have been revealed to explain how processing of amyloidogenic proteins can drive tumor growth and promote drug resistance. The mechanisms by which BACE2 activity affects cancer growth seem to be deeply linked to the ability of this protease to process amyloidogenic proteins that in turn modulate TME.

In particular, the BACE1/2 dependent maturation of amyloidogenic proteins such as APP or PMEL, has been shown to affect TME cells behavior and to act both in paracrine and autocrine way driving tumor proliferation and progression [44, 48, 69]. Importantly, it has also to be noted that β-secretases, besides processing amyloidogenic proteins, have a broad range of substrates; for example, it has been shown that BACE1 can process IL6R, inducing macrophages polarization towards a pro-tumoral phenotype [73].

Moreover, BACE2 can affect intracellular pathways enhancing tumor proliferation via the upregulation of the NFkB pathway [46] or through Ca^2+^ intracellular accumulation [74].

It has to be noted however, that the overexpression of BACE2 in cancer tissues seems to be quite widespread justifying further studies to discover other pathways implicated in the development and progression of the disease.

All these evidences highlight the importance of BACE1/2 targeting in cancer to counteract tumor growth and progression. Different compounds, that efficiently reduce BACE1/2 activity and amyloid processing are available. Many of them were tested in clinical trials for the treatment of Alzheimer's disease, where they reduce amyloid plaque in the extracellular space but they were not able to reduce the cognitive defect characteristic of the disease, thus failing to pass Phase III clinical trials [77].

However, these compounds specifically reduce the BACE1/2 activity causing a decrease of amyloid aggregates, and they could therefore be exploited in the treatment of different types of tumors where BACE1/2 derived amyloid aggregates have been shown to play a fundamental role (Table 1).

**Table 1:** Involvement of BACE1/2 in different types of cancer where the inhibition of these enzymes curbed tumor proliferation and/or progression.

Cancer	BACE1 inhibition	BACE2 inhibition	BACE1/2 inhibition
**Melanoma**	-	Decreased proliferation of metastatic melanoma cells [44] and tumor volume in ocular melanoma [74]	Decreased proliferation and enhance chemosensitivity of melanoma cells [44]; reduced brain metastasis [69] and reduce tumor volume [48]
**Pancreas**	-	-	Reduced cancer cell proliferation [49]
**Glioma**	Reduced tumor volume and synergized with radiotherapy [73]	Reduced cancer cell proliferation, migration and invasion [46]; reduced tumor volume and enhanced sensitivity to radiotherapy [45]	-

One limitation of the studies described in this review is that only seldom it has been distinguished between BACE1 or 2 specific activity, raising some concerns about possible spurious effects. Indeed, even if BACE2 seems to be the β-secretase mainly involved in cancer, the availability of BACE2 specific inhibitors is problematic due to the high grade of homology with BACE1. Only in recent years, BACE2 specific inhibitors have been synthetized to be used in diabetic patients [78], but up to now no clinical trials are available on these compounds.

BACE2 specific inhibition have also the advantage to show fewer side effects compared to BACE1/2 dual inhibition, as the only documented one is reversible hypopigmentation due to impairment of melanosome maturation [40].

Further, it would be interesting to define which are the mechanisms that drive the over-expression of BACE2 in different cancer subtypes looking at SNP (single nucleotide polymorphism) and CNV (copy number variation) as it was reported for patients affected by Alzheimer’s disease [79].

In conclusion, it is becoming evident that β-secretases play a crucial role in cancer biology. Further studies are necessary to dissect the role of BACE1 and 2 and of their targets to better characterize the mechanisms by which BACE1/2 drive cancer development and progression with the final goal to lay the foundation for future therapeutic strategies.

## Data Availability

Not applicable.

## Abbreviations

BACE1: β-site APP cleaving enzyme 1
BACE2: β-site APP cleaving enzyme 2
ER: Endoplasmic Reticulum
SP: Signal Peptide
APP: amyloid-beta precursor protein
sAPPβ: soluble amyloid precursor proteinβ
Aβ: Amyloid Beta
APLP2: Amyloid-like protein 2
Nrg1: Neuregulin 1
Jag1: Jagged-1
TMEM27: Trimeric intracellular cation channel 27
T2D: type 2 diabetes
hIAPP: human Islet amyloid polypeptide
PMEL: premelanosome protein
AD: Alzheimer's Disease
GTEx: Genotype-tissue expression
TCGA: The Cancer Genome Atlas
GEPIA: Gene Expression profiling interactive analysis
CHOL: Cholangiocarcinoma)
COAD: Colon adenocarcinom
GBM: Glioblastoma
KICH: Kidney Chromophobe
OV: Ovarian serous cystadenocarcinoma
PAAD: Pancreatic adenocarcinoma
READ: Rectum adenocarcinoma
SKCM: Skin Cutaneous Melanoma
TME: Tumor Microenvironment
ECM: Extracellular matrix
CSF1: colony stimulating factor 1
IL6: Interleukin-6
IL10: Interleukin-10
TGFβ: transforming growth factor-β
VEGF: vascular endothelial growth factor
CAFs: cancer associated fibroblasts
NETs: Neutrophil Extracellular Traps
YAP: Yes associated protein
TAM: Tumor associated macrophages
sIL6R: soluble IL-6 receptor
pTAM: pro-tumoral macrophages
sTAM: tumor suppressive macrophages
NF-κB: nuclear factor kappa-light-chain-enhancer of activated B cells
IKKβ: inhibitor of nuclear factor kappa-B kinase subunit beta
IKBα: nuclear factor of kappa light polypeptide gene enhancer in B-cells inhibitor, alpha
TMEM38B: Trimeric intracellular cation channel 38B
PI3K: Phosphoinositide 3-kinases
SNP: single nucleotide polymorphism
CNV: copy number variation
